# Detection of hysteroscopic fluid in the pouch of Douglas: a prospective cohort study about the predictability of bilateral tubal occlusion

**DOI:** 10.1007/s00404-021-05993-0

**Published:** 2021-02-24

**Authors:** Marlene Hager, Johannes Ott, Christian Göbl, Iris Holzer, Rudolf Seemann, Christine Kurz, John Preston Parry

**Affiliations:** 1 Clinical Division of Gynecological Endocrinology and Reproductive Medicine, Medical University of Vienna, Spitalgasse 23, 1090 Vienna, Austria; 2 Clinical Division of Obstetrics and Feto-Maternal Medicine, Department of Obstetrics and Gynecology, Medical University of Vienna, Vienna, Austria; 3 Department of Oral and Maxillofacial Surgery, Medical University of Vienna, Vienna, Austria; 4 Parryscope® and Positive Steps Fertility, Madison, MS USA; 5 Department of Obstetrics and Gynecology, University of Mississippi Medical Center, Jackson, MS USA

**Keywords:** Fallopian tubes, Tubal patency, Chromopertubation, Laparoscopy, Female infertility, Hysteroscopy

## Abstract

**Purpose:**

To determine whether an increase in cul de sac (CDS) fluid after hysteroscopy is predictive of tubal patency.

**Methods:**

In a prospective clinical cohort study, 115 subfertile women undergoing laparoscopic and hysteroscopic surgery at the Medical University of Vienna were invited to participate. The primary outcome was determining whether an increase in fluid in the pouch of Douglas was reflective of unilateral or bilateral tubal patency. Vaginal sonography before and after hysteroscopy was performed to detect fluid in the pouch of Douglas, directly followed by laparoscopy with chromopertubation.

**Results:**

Laparoscopic chromopertubation revealed bilateral Fallopian tube occlusion in 28 women (24.3%). Twenty-seven/40 patients (67.5%) with no fluid shift had bilateral occlusion during the consecutive laparoscopy (*p* < 0.001). One/75 patients (1.3%) showing a fluid shift had bilateral occlusion (sensitivity of a present fluid shift for uni- or bilateral patency 85.1%, 95% CI: 81.7–99.9, specificity: 96.4%, 95% CI: 75.8–91.8). Intracavitary abnormalities (odds ratio, OR, 0.038; *p* = 0.030) and adhesions covering one or both tubes (OR 0.076; *p* = 0.041) increased the risk for a false abnormal result, i.e., uni- or bilateral tubal patency despite the lack of a fluid shift.

**Conclusion:**

When CDS fluid does not change after hysteroscopy, this is a sensitive test for tubal occlusion and further testing may be warranted. However, if there is an increase in CDS fluid after hysteroscopy, particularly for a patient without fluid present prior, this is both sensitive and specific for unilateral or bilateral tubal patency.

## Introduction

Hysteroscopy is known as the gold standard for intrauterine evaluation [1, 2] and can routinely be performed in office settings [2–4]. Conversely, the gold standard for tubal evaluation through laparoscopic chromopertubation requires general anesthesia [5]. If diagnostic hysteroscopy can accurately be used to assess tubal patency, particularly through office settings, this can lower cost and enhance patient satisfaction by avoiding the operating room and incisions [2, 6]. The various reported methods have recently been reviewed [7]. Two of them were evaluated in a recent randomized controlled trial by our group [8]–the Parryscope technique where air bubbles are introduced into the uterine cavity and are observed whether they traverse the tubal ostia, [2, 7–9] and the Flow technique. Although the Parryscope was shown superior to the Flow method [8] and achieved high overall accuracy [8, 9], use of sonography to detect post-hysteroscopy shifts of cul de sac (CDS) fluid was previously described for supplementing the Parryscope technique [9]. Notably, above 90% of women with tubal occlusion had no considerable shift from pre- to post-hysteroscopy [9]. In contrast, Peregrin-Alvarez et al. conducted a prospective observational study, wherein they claimed that all women with at least one laparoscopically proven patent tube showed a pre- to post-hysteroscopic fluid shift [10].

Neither of these studies, in describing finding studies, took all patients to the operating room. For the Peregrin-Alvarez study, no patients went to the operating room, and for the Parry, et al., study, though an association was described between occlusion at surgery and a lack of increase in CDS fluid after hysteroscopy, the associated sensitivity and specificity was not published. Since there are few publications on this issue, our research goal was to better understand how tubal patency affects CDS fluid shifts through a prospective cohort study.

## Materials and methods

### Patient population

In a prospective, monocentral, clinical cohort study, 115 women ages 18–44 were enrolled between March 2018 and November 2019. Participants were undergoing a fertility evaluation at the Clinical Division of Gynecologic Endocrinology and Reproductive Medicine of the Medical University of Vienna, Austria. All participants signed written affirmation of informed consent prior to a combination of hysteroscopy and laparoscopy with chromopertubation. Vaginal sonography was performed before and after the hysteroscopy. Patients who had already removed one or both of their Fallopian tubes were excluded from participation in the study. The study was approved by the Institutional Review Board of the Medical University of Vienna (IRB number 1096/2018). The dataset is available online (https://data.mendeley.com/datasets/zf3hp5myyg/1).

### Surgical technique

All surgical procedures were conducted under general anesthesia and either directly performed or supervised by experts in infertility surgery [6, 8, 11]. A forward-oblique 30° hysteroscope (Karl Storz GmbH & Co KG, Tuttlingen, Germany; sheath diameter: 5 mm) was used for diagnostic hysteroscopy. With an intravenous solution of 0.9% sterile saline, tubing with a drip chamber, and a reusable IV pressure bag, continuous inflow was generated [8]. Intrauterine length was measured from the uterine fundus to the external os of the cervix.

A “Spackmann” uterine manipulator with clamp fixation and a rubber cone with a diameter of 18 mm (Reference number 1264; WISAP^®^ Medical Technology GmbH Brunnthal/Hofolding, Germany) was placed through the cervix before laparoscopy with chromopertubation.

With laparoscopy, the uterine manipulator was placed 1 cm from the uterine fundus for each patient [6, 8]. During the laparoscopy, thorough inspection of the pelvis, internal genitalia, and liver region was conducted and the subsequent chromopertubation was performed using a 50-mL syringe with a dilute solution of indigo carmine blue dye (Amino AG Gebersdorf, Switzerland) through the uterine manipulator. Parameters recorded include patency of the Fallopian tubes, the volume of dye utilized with chromopertubation and an assessment of the subjectively required pressure to achieve tubal patency [6, 8].

### Sonographic assessment of fluid in Douglas pouch

Vaginal sonography with an Aloka Prosound 6 ultrasound machine (Wiener Neudorf, Austria) was performed in all patients pre-hysteroscopy and post-hysteroscopy to assess shifts in fluid volume for the pouch of Douglas. The fluid pockets located in the vaginal rear fornix were measured, as previously characterized [12]. Specifically, the longitudinal length of the fluid pocket was measured in the sagittal field, whereas the coronal field was used to measure the diagonal dimension and depth of the fluid pocket. The following ellipsoid formula was used to calculate the fluid volumes in the peritoneal cavity: V (volume) = ¼ d1 (longitudinal measurement) *d2 (transverse measurement) *d3π (perpendicular measurement)/6.

### Parameters analyzed

The parameters assessed were documented by the senior surgeon using a prospective case report form. We considered the presence of a sonographic fluid shift in Douglas pouch before and after the hysteroscopy to be the main outcome parameter. These findings were validated by laparoscopic chromopertubation. Furthermore, hysteroscopically diagnosed abnormalities in the uterine cavity, tubal patency in chromopertubation (for each side) and information about further laparoscopic surgical interventions were considered additional outcome parameters. Outcomes were always supervised by a study member who was not part of the surgical team for each respective operation. In addition, tracked parameters include full uterine length, patient’s age and body mass index (BMI), the type of infertility (primary versus secondary), the surgical indication, and the menstrual cycle day at surgical date.

### Sample size calculation

A similar study about transvaginal sonography for the detection of hysteroscopic fluid in Douglas pouch assessed a group with lower risk for surgical pathology [10] relative to those seen in our department. In a previous study conducted by our own group, no post-hysteroscopy cul de sac fluid was identified in 94.4% of cases with bilateral tubal occlusion, whereas in cases with unilateral occlusion or bilateral tubal patency, only 5.3% had no post-hysteroscopy fluid detected [13]. Since our patients had a high rate of pelvic pathology, such as hydrosalpinges, adhesions, moderate to severe endometriosis, and more, we assumed the predictive value of bilateral tubal occlusion to be smaller. We, therefore, calculated sample size for the absence of fluid in 90% relative to 30%, respectively. An alpha of 0.01 and a power of 95% required 23 patients per study arm after a Fleiss-correction. Our previous experience had shown 20% of our patient population had bilateral tubal occlusion [13]. This correlated with enrollment of 115 patients (23 patients with bilateral tubal occlusion and 92 patients with unilateral occlusion or bilateral tubal patency).

### Statistical analysis

The AKIM software (SAP-based patient management system at the Medical University of Vienna) was used for data acquisition, after data were collected and documented via prospective case report form and an Excel^®^-table. Data analyses were conducted using SPSS software 24.0 (SPSS Inc, 1989–2018). Numerical data with a normal distribution were reported as mean and standard deviations, numerical data without a normal distribution as median and interquartile range (IQR), while categorical data (primary/secondary sterility, indication for surgical intervention, abnormalities in the uterine cavity in hysteroscopy, tubal patency in chromopertubation, information about further laparoscopic interventions during the operation) were reported as number and percentage.

Differences between groups in categorical parameters were tested using the Chi square test or the Fisher’s exact test. Overall accuracy, sensitivity, specificity, positive and negative predictive values including the 95% confidence intervals (95% CI) were calculated for presence of a sonographic cul de sac fluid shift for the prediction of bilateral tubal occlusion. Risk factors for false-normal results were evaluated. For this, a multivariate binary regression model was used. This analysis used odds ratios with 95% confidence intervals (95% CI), the Wald-test, and the likelihood ratio (LR) test. Differences were considered significant if *p* < 0.05.

## Results

Patient characteristics are provided in Table 1. In 11 women (9.6%), a cervical stricture was identified and treated with Hegar dilators. Including those requiring dilation, the uterus was successfully accessed via hysteroscopy for all participants. A normal cavity was seen in 92 patients (80.0%), whereas 9 (7.8%), 2 (1.7%), 1 (0.9%), and 13 (11.3%) women revealed endometrial polyps, myomas FIGO type 0–2, intrauterine adhesions, and uterine malformations, respectively. The view in diagnostic hysteroscopy was rated as clear in 88 (76.5%) and as cloudy in 27 (23.5%) cases. Intrauterine adhesiolysis was performed with the tip of the diagnostic hysteroscope. After diagnostic hysteroscopy, a dilatation and curettage was performed for removal of polyps in 9 cases (7.8%) and an operative hysteroscopy with a hysteroscope of a larger diameter was performed due to myomas or a septate uterus in 12 women (10.4%). In three patients, the uterine malformation was a bicornuate uterus for which surgical intervention was not warranted. Uterine length ranged from 5 to 12 cm (7.3 ± 1.1 cm).

**Table 1:** Basic patient characteristics

Age (years)*	32.5 ± 5.2
BMI (kg/m^2^)*	24.3 ± 4.8
Phase of menstrual cycle at the day of surgery^#^	
Follicular phase	102 (88.7)
Luteal phase	13 (11.3)
Primary sterility^#^	86 (74.8)
Indication for surgery^#+^	
Endometrial polyp	4 (3.5)
Suspicion of endometriosis	51 (44.3)
Suspicion of tubal factor	21 (18.3)
Laparoscopic ovarian drilling	11 (9.6)
Uterine malformation	14 (12.2)
Ovarian cyst	36 (31.3)
Myoma	17 (4.8)
Otherwise unexplained infertility	6 (5.2)

Data are provided as * mean ± standard deviation or ^#^ number (frequency); ^+^ multiple mentions are possible

In subsequent laparoscopic chromopertubation, there were 152 (66.1%) patent Fallopian tubes (127 with normal, i.e., low chromopertubation pressure, and 25 with increased chromopertubation pressure) and 78 (33.9%) occluded Fallopian tubes (66 proximal and 12 distal occlusions). Chromopertubation revealed a unilateral tubal occlusion in 22 (19.1%) and bilateral tubal occlusion in 28 (24.3%) women. Notably, in 11/22 (50.0%) patients with unilateral occlusion, high chromopertubation pressure was necessary to achieve patency. For the 50 women with unilateral or bilateral Fallopian tube occlusion, 26 (52%) related to endometriosis and/or hydrosalpinges, while neither of these were identified in 24 (48%).

To detect fluid in the pouch of Douglas, vaginal sonography was conducted before and after hysteroscopy. Preoperatively, cul de sac fluid was identified in 15 patients (13.0%). In these women, a median amount of 3.4 mL (IQR 2.5–6.0 mL) was found. This was more often the case in the luteal phase (6/7, 85.7%) compared to the follicular phase (9/93, 9.7%; *p* = 0.002). After hysteroscopy, cul de sac fluid was found in 80/115 patients (69.6%), with a median volume of 15.5 mL (IQR 5.3–30.0 mL). However, a clearly visible fluid shift was identified for 75 women (65.2%). Women with bilateral tubal patency revealed a significantly higher increase in cul de sac fluid than women with unilateral tubal occlusion and women with bilateral tubal occlusion (*p* < 0.001; Table 2).

**Table 2:** Comparison of sonographic pre- to post-hysteroscopic fluid shifts according to the number of patent Fallopian tubes

	Bilateral tubal patency (*n* = 65)	Unilateral tubal occlusion (*n* = 22)	Bilateral tubal occlusion (*n* = 28)	*p*
Visible fluid shift^a^	62 (95.4)	12 (54.5)	1 (3.6)	< 0.001
Fluid shift (mL)^b^	18.0 (8.0;30.0)	5.5 (0.0;5.3)	0 (0;0)	< 0.001

^a^Nominal variable, provided in *n* (%)

^b^Continuous variable, provided in median (IQR) ± standard deviation

As demonstrated in Table 3A, the presence of a fluid shift is predictive of unilateral or bilateral patency in a statistically significant manner (*p* < 0.001). Twenty-seven of 40 patients (67.5%) for whom no fluid shift was identified had bilateral occlusion during the subsequent laparoscopy, whereas this was the case for only 1/75 patients (1.3%) showing a visible fluid shift, which results in a NPV of 67.5% (95% CI: 50.9–81.4) and a PPV of 98.7% (95% CI: 92.8–100.0%). The one patient with a sonographic fluid shift despite bilateral tubal occlusion at laparoscopy may have been a false positive with chromopertubation. She was a 34 years old woman who underwent the operation for primary infertility, suspicion of an endometrial polyp and suspicion of endometriosis (both of which were confirmed). During laparoscopy, a bilateral proximal tubal occlusion was diagnosed as well as endometriosis of rASRM stage 3 (without endometriomas). The sonographically measured volume of fluid in the pouch of Douglas had increased from 2.4 mL pre-hysteroscopy to 15.0 mL post-hysteroscopy. About 2 months after the operation, a hysterosalpingo-contrast-sonography was performed in an outpatient setting and revealed bilateral tubal patency, suggesting that chromopertubation had improperly identified bilateral occlusion.

**Table 3:** Testing for bilateral Fallopian tube occlusion (A) and bilateral Fallopian tube patency (B) with pre- and post-hysteroscopy sonography to assess fluid shifts

A	Laparoscopic chromopertubation			p*	< 0.001
	Unilateral or bilateral patency	Bilateral occlusion	Sum	Sensitivity (%)^#^	85.1 (75.8; 91.8)
Fluid shift					
Present	74	1	*75*	Specificity (%)^#^	96.4 (81.7; 99.9)
Absent	13	27	*40*	PPV (%)^#^	98.7 (92.8; 100.0)
Sum	*87*	*28*	*115*	NPV (%)^#^	67.5 (50.9; 81.4)

B	Laparoscopic chromopertubation			p*	< 0.001
	Bilateral patency	Unilateral or bilateral occlusion	Sum	Sensitivity (%)^#^	95.4 (87.1; 99.0)
Fluid shift					
Present	62	13	*75*	Specificity (%)^#^	74.0 (59.7; 85.4)
Absent	3	37	*40*	PPV (%)^#^	82.7 (72.2; 90.4)
Sum	*65*	*50*	*115*	NPV (%)^#^	92.5 (79.6; 98.4)

*PPV* positive predictive value; *NPV* negative predictive value

*P* values were calculated either using the Fisher’s exact test

^#^Results are provided with the 95% confidence interval

We also tested whether the presence of a fluid shift would bilateral tubal patency (Table 3B). Of the 40 women without a fluid shift, 37 (92.5%) had either unilateral or bilateral tubal occlusion, in contrast to 13/75 (17.3%) patients with a sonographic fluid shift (*p* < 0.001). In other words, of 40 women without a fluid shift, only 3 (7.5%) had bilateral tubal patency. The associated sensitivity was 74% (95% CI: 59.7–85.4%) and specificity 95.4% (95% CI: 87.1–99.0%). Of the ten women with unilateral occlusion and without a fluid shift, at laparoscopy, seven (70.0%) revealed peritubal adhesions on the patent side.

In addition, we did subset analysis in the 40 patients for whom no fluid shift had been seen and compared those with (*n* = 27, 67.5%) to those without (*n* = 13, 32.5%) bilateral Fallopian tube occlusion (Table 4). The 13 women with no fluid shift but bilateral patency were much more likely to have intracavitary abnormality (OR 0.038, 95% CI: 0.002–0.726; *p* = 0.030) or adhesions covering one or both tubes (OR 0.076, 95% CI: 0.006–0.902; *p* = 0.041). When chromopertubation was performed demonstrating patency, it was after correction of intrauterine pathology in 7/13 women (53.8%), whereas in 6/13 women (46.2%) with neither a fluid shift nor bilateral Fallopian tubal occlusion, neither intracavitary abnormalities nor adhesions covering one or both tubes were found.

**Table 4:** Women without a visible fluid shift in the pouch of Douglas: comparison between patients with and without bilateral tubal occlusion

	Bilateral tubal occlusion (*n* = 27)	Uni- or bilateral tubal patency (*n* = 13)	Multivariate analysis		
			Adjusted OR (95% CI)^a^	*P* (Wald’s test)	*P* (LR test)^b^
Age (years)^c^	34.4 ± 4.4	34.6 ± 4.2	1.053 (0.838; 1.322)	0.195	0.659
Body mass index (kg/m^2^)^c^	25.2 ± 4.5	24.2 ± 4.4	1.077 (0.787; 1.474)	0.216	0.642
Follicular phase^d^	23 (85.2)	11 (84.6)	0.812 (0.010; 68.664)	0.008	0.927
Secondary infertility^d^	7 (25.9)	5 (38.5)	1.806 (0.155; 21.059)	0.222	0.637
Any intracavitary abnormality^d,e^	2 (7.4)	6 (46.2)	0.038 (0.002; 0.726)	4.724	*0.030*^f^
Uterine malformation^d,e^	0	3 (23.1)	0 (0; –)	0.000	0.999
Ovarian cyst^d,e^	6 (22.2)	3 (23.1)	0.290 (0.021; 3.942)	0.864	0.353
Endometriosis^d,e^	10 (37.0)	5 (38.5)	0.592 (0.055; 6.636)	0.187	0.665
Uni- or bilateral hydrosalpinx^d,e^	3 (11.1)	3 (23.1)	0.130 (0.002; 7.501)	0.972	0.324
Adhesions covering one or both tubes^d,e^	11 (40.0)	9 (69.2)	0.076 (0.006; 0.902)	4.168	*0.041*^f^
Myoma(s)^d,e^	5 (18.5)	2 (15.4)	0.471 (0.028; 7.804)	0.277	0.599
Constant	–	–	1.676 (–; –)	0.009	0.926

^a^OR (95% CI) = odds ratio (95% confidence interval)

^b^*LR test* = likelihood ratio test

^c^Continuous variable, provided in mean ± standard deviation

^d^Nominal variable, provided in *n* (%)

^e^Final diagnosis during combined hysteroscopy/laparoscopy, multiple entries allowed

^d^Italic letters indicate statistical significance. “Any intracavitary abnormality” includes: endometrial polyps, intrauterine adhesions, myoma(s) FIGO 0–2, and uterine malformations

## Discussion

Comparing pre- to post-hysteroscopic cul de sac fluid using vaginal sonography allows the prediction of bilateral Fallopian tube conclusion in a reliable manner. An absent fluid shift is associated with bilateral tubal occlusion with a sensitivity of 96.4% and a NPV of 85.1%. Moreover, given that follow-up suggests that the one outlier may have related to false-positive occlusion with chromopertubation, one could argue the sensitivity to be 100%. In short, an increase in post-hysteroscopy cul de sac fluid is reassuring that at least one or both Fallopian tubes are open, while the lack of a change in cul de sac fluid warrants suspicion for tubal occlusion.

A few studies have already explored this issue. In 2009, Yildizhan et al. compared pre- and post-hysteroscopic fluid shifts for 56 women relative to hysterosalpingography and found a sensitivity and a PPV of 77.8% and 87.5%, respectively [14]. However, assessments of accuracy may have been hindered by the authors not using the gold standard of laparoscopy for a true determination of patency. When later reports compared sonographic fluid shifts to laparoscopic chromopertubation, a higher degree of sensitivity was observed in two reports with 94% (Habibaj et al.) [15] and 92% (Parry et al.) [9]. These findings are similar to our results, which also relied on laparoscopic controls. Of note, both of these relied on diagnostic hysteroscopy. However, longer hysteroscopies through an operative component in the present study may have influenced fluid shifts. Women with bilateral tubal patency showed the highest pre-hysteroscopy to post-hysteroscopy increase in cul de sac fluid (median 18.0 mL), followed by women with unilateral patency (median 5.5 mL) and women with bilateral occlusion (median 0.0 mL; *p* < 0.001; Table 2). This is not surprising and is consistent with previous observation [14, 16]. In addition, these differences in cul de sac volume were surprisingly similar to those reported by Yildizhan et al. (6.9 ± 2.7 mL versus 4.2 ± 0.9 mL versus 1.1 ± 0.7 mL, respectively) [14]. It should be expected that future studies will show variations in absolute accumulated cul de sac fluid volume. The reason is that final volumes are dependent on pre-hysteroscopy volume (where one can see variation in repeated measures for patients with high initial volume) and on the duration of hysteroscopy, as the faster the procedure, the more limited the opportunity for fluid to accumulate in the cul de sac.

Accordingly, cul de sac fluid shifts might not only predict bilateral but also unilateral tubal occlusion. As seen in Table 3, women with unilateral occlusion were likely to have no detectable increase in cul de sac fluid. With seven out of ten (70%) of women with unilateral patency having adhesions ipsilateral to the patent tube, one must account for technical patency at chromopertubation not always accounting for intraluminal damage that does not fully obstruct the tube. Stenosis of the lumen without occlusion through previous intraluminal damage (such as from pelvic inflammatory disease) that also results in ipsilateral adhesions may hinder distention media from hysteroscopy traversing the tube even if it happens to be technically open at chromopertubation. This overlaps with previous findings that patients requiring high pressure to demonstrate patency at hysterosalpingography have lower pregnancy rates than those where dye readily traverses the Fallopian tube [16]. Women without an increase in cul de sac fluid after hysteroscopy likely warrant additional scrutiny, particularly if there are risk factors for potential tubal disease.

Attention is also warranted for “false abnormal results,” where for some patients fluid did not accumulate in the cul de sac despite the Fallopian tubes being technically open. Many of these women had intrauterine pathology and particularly adhesions. If the tube were occluded through the pathology and the moment the pathology was removed, hysteroscopy was ceased, this would be consistent with limited opportunity for fluid to accumulate. Though not all intrauterine abnormalities will fully occlude a tube, the correlation between limited accumulation of fluid post-hysteroscopy with both tubal occlusion and intrauterine pathology still means that these patients warrant evaluation.

However, it must be assumed that the minimum rate of tubal spasm in the course of laparoscopic chromopertubation was 0.9% (1/115) in our patient population. Thus, for the first time, tubal spasm during laparoscopic chromopertubation can be defined and diagnosed, at least those cases that occur after hysteroscopy. Accordingly, we believe that performing a vaginal ultrasound directly before and after hysteroscopy might also be a valuable tool in infertile women who undergo hysteroscopy and subsequent laparoscopy with chromopertubation. It has to be noted that this additional procedure can be performed easily and is time efficient.

Given reasonable sensitivity, specificity, positive, and negative predictive values, the presence and absence of fluid shifts in the pouch of Douglas can provide valuable insight for tubal patency in patients undergoing hysteroscopy during a procreative workup. Moreover, cul de sac fluid shifts can be combined with observational (such as the flow technique [6]) and interventional (the Parryscope technique [9] and tubal cannulation with dye infusion [17]) information acquired during diagnostic hysteroscopy. The flow and the Parryscope techniques are the fastest and the lowest cost of the three approaches, and the latter is more accurate than the former when they were directly compared in a randomized controlled trial [8].

The strength of this study is that all included women underwent laparoscopic chromopertubation which allowed comparison of the sonographic results to the gold standard. Moreover, additional data about endometriosis, adhesions and other intraabdominal abnormalities could be evaluated. However, a few study limitations have to be mentioned. One might argue that the rate of women with a cloudy view in hysteroscopy was high (23.5%). However, a previous study has demonstrated that this finding was associated with bilateral tubal occlusion [6] which was comparably frequent in our dataset (24.3%). Moreover, the present study did not precisely quantify laparoscopic pressures necessary to achieve tubal patency. This might be of relevance given that high-pressure patency is associated with lower fecundity than when it is observed with lower pressure [7, 16]. However, this constraint is common to practically all publications on tubal patency. A similar limitation in this study and common to tubal patency research is that patency does not equate with tubal ciliary function. Also of note, hysteroscopy was performed before laparoscopy, where for longer operative hysteroscopies, myometrial edema could have occurred creating false occlusion at laparoscopy when the tubes would normally be patent. This potential limitation, however, does not seem to have affected the results. The reason relates to having only one case where patency was suggested by cul de sac fluid increases despite having occlusion at laparoscopy (and then patency on subsequent sonosalpingography), and this case only had brief hysteroscopy (with a net increase of 12.5 mL for cul de sac fluid). Finally, there are likely contexts where the accuracy of this approach may be limited. These likely include high CDS fluid volumes prior to hysteroscopy, where repeated measures lead to variability and patients requiring significant cervical dilation, which may promote transient uterine spasm, decreasing tubal outflow. Further research will allow for better subset analysis in these circumstances.

## Conclusion

Sonographically observing pre- to post-hysteroscopic fluid shifts in the pouch of Douglas offers sensitive and specific information about tubal patency and occlusion, which can be useful in counseling patients with subfertility. The absence of a fluid shift can be suggestive of both unilateral and bilateral tubal occlusion, where women with these findings likely should undergo additional tubal patency assessment. In addition, women with increases in cul de sac fluid volume, particularly if they intend to try to conceive naturally, can be reassured that they likely have unilateral or bilateral patency. Moreover, when patients have indeterminate results through direct hysteroscopic assessment, such as the flow and Parryscope techniques, concurrent ultrasound can often still allow for enough guidance as to make decisions on next steps, particularly when accounting for known risk factors. Particularly when combining hysteroscopy and sonography in the office, this minimally invasive approach to the fertility evaluation may offer meaningful information for patients.

## Data Availability

Data will be provided if necessary.

## References

[1] Campo R, Meier R, Dhont N, Mestdagh G, Ombelet W (2014). Implementation of hysteroscopy in an infertility clinic: the one-stop uterine diagnosis and treatment. Facts Views Vision ObGyn.

[2] Parry JP, Isaacson KB (2019). Hysteroscopy and why macroscopic uterine factors matter for fertility. Fertil Steril.

[3] Di Spiezio SA, Taylor A, Tsirkas P, Mastrogamvrakis G, Sharma M, Magos A (2008). Hysteroscopy: a technique for all? Analysis of 5000 outpatient hysteroscopies. Fertil Steril.

[4] Guida M, Di Spiezio SA, Acunzo G, Sparice S, Bramante S, Piccoli R, Bifulco G, Cirillo D, Pellicano M, Nappi C (2006). Vaginoscopic versus traditional office hysteroscopy: a randomized controlled study. Hum Reprod.

[5] Godinjak Z, Idrizbegović E (2008). Should diagnostic hysteroscopy be a routine procedure during diagnostic laparoscopy in infertile women?. Bosnian J Basic Med Sci.

[6] Ott J, Hager M, Nouri K, Marschalek J, Kurz C (2020). Assessment of tubal patency: a prospective comparison of diagnostic hysteroscopy and laparoscopic chromopertubation. J Mini Invas Gynecol.

[7] Hager M, Simek IM, Promberger R, Ott J (2019). The role of diagnostic hysteroscopy in the evaluation of Fallopian tube patency: a short review. Geburtshilfe Frauenheilkunde.

[8] Hager M, Ott J, Holzer I, Seemann R, Kurz C, Parry JP (2020). Hysteroscopic assessment of tubal patency: a randomized comparison between the flow and Parryscope techniques. J Mini Invas Gynecol.

[9] Parry JP, Riche D, Aldred J, Isaacs J, Lutz E, Butler V, Shwayder J (2017). Proximal tubal patency demonstrated through air infusion during flexible office hysteroscopy is predictive of whole tubal patency. J Mini Invas Gynecol.

[10] Peregrin-Alvarez I, Roman R, Christiansen ME, Morris J, Detti L (2019). Fluid deficit calculation at hysteroscopy in patients with and without tubal occlusion: could consideration of tubal passage change safety limits?. Fertil Steril.

[11] Hager M, Wenzl R, Riesenhuber S, Marschalek J, Kuessel L, Mayrhofer D, Ristl R, Kurz C, Ott J (2019). The prevalence of incidental endometriosis in women undergoing laparoscopic ovarian drilling for clomiphene-resistant polycystic ovary syndrome: a retrospective cohort study and meta-analysis. J Clin Med.

[12] Siftar JP, Sobocan M, Takac I (2018). The passage of fluid into the peritoneal cavity during hysteroscopy in pre-menopausal and post-menopausal patients. J Obstet Gynaecol.

[13] Promberger R, Simek IM, Nouri K, Obermaier K, Kurz C, Ott J (2018). Accuracy of tubal patency assessment in diagnostic hysteroscopy compared with laparoscopy in infertile women: a retrospective cohort study. J Mini Invas Gynecol.

[14] Yildizhan B, Durmusoglu F, Uygur M, Erenus M (2009). A new technique for the diagnosis of fallopian tube patency by using hysteroscopy with ultrasound compared with hysterosalpingography in infertile women. Arch Gynecol Obstet.

[15] Habibaj J, Kosova H, Bilali S, Bilali V, Qama D (2012). Comparison between transvaginal sonography after diagnostic hysteroscopy and laparoscopic chromopertubation for the assessment of tubal patency in infertile women. J Clin Ultrasound.

[16] Karande VC, Pratt DE, Gleicher N (1996). The assessment of tubal functional status by tubal perfusion pressure measurements. Hum Rep Update.

[17] Török P, Major T (2012). Accuracy of assessment of tubal patency with selective pertubation at office hysteroscopy compared with laparoscopy in infertile women. J Mini Invas Gynecol.

